# Smoking-driven systemic inflammation elevates mortality risk in hypertensive patients: A cross-sectional study using insights from NHANES 1999–2018

**DOI:** 10.18332/tid/214125

**Published:** 2026-01-31

**Authors:** Tingting Wu, Chufan Ren, Chenhan Wei, Yang Yu, Tiancheng Jin, Yihang Wang, Hongde Chen

**Affiliations:** 1 Center for Geriatric Medicine, Key Laboratory of Alzheimer’s Disease of Zhejiang Province, The First Affiliated Hospital and Institute of Aging, Wenzhou Medical University, Wenzhou, China; 2 Wenzhou Medical University, Wenzhou, China; 3 Department of General Surgery, Chifeng Hospital, Chifeng, China; 4 Department of Urology, The First Affiliated Hospital of Wenzhou Medical University, Wenzhou, China; 5 Zhejiang Key Laboratory of Intelligent Cancer Biomarker Discovery and Translation, First Affiliated Hospital, Wenzhou Medical University, Wenzhou, China; 6 Central Laboratory, The First Affiliated Hospital of Wenzhou Medical University, Wenzhou, China

**Keywords:** smoking, hypertension, systemic inflammation, mortality, National Health and Nutrition Examination Survey

## Abstract

**INTRODUCTION:**

Existing evidence on the association between smoking and hypertension (HTN) remains conflicting, and the potential role of systemic inflammation in mediating smoking-related mortality among hypertensive patients is poorly understood. This study aimed to investigate the association between smoking status, smoking volume, and HTN risk in a large, nationally representative sample. Furthermore, we sought to determine whether systemic inflammation, measured by the systemic inflammation index (SII), mediates the association between smoking and all-cause mortality in hypertensive individuals.

**METHODS:**

This cross-sectional, pooled secondary data analysis study utilized data from 10 cycles of the National Health and Nutrition Examination Survey (NHANES) from 1999 to 2018. Data on smoking, covariates, and hypertension status were collected through standardized interviews, questionnaires, and laboratory/physical examinations. A total of 28967 participants were included after excluding those with incomplete data. Propensity score matching (PSM) analysis was employed to adjust for confounding factors such as age, gender, BMI, race, and other sociodemographic variables. Logistic regression and restricted cubic spline regression were used to assess the dose-response relationship between smoking and HTN. Mediation analysis was performed to evaluate the role of systemic inflammation, as measured by the systemic inflammation index (SII), in the increased mortality risk among hypertensive smokers.

**RESULTS:**

Smoking significantly increased the likelihood of HTN after adjusting for confounders (adjusted odds ration, AOR=1.18; 95% CI: 1.10–1.27). A dose-response relationship was observed, with individuals smoking >30 cigarettes/day having the highest likelihood of HTN (AOR=1.37; 95% CI: 1.07–1.75). PSM analysis confirmed these findings, showing a significant increase in HTN prevalence among smokers (p=0.045). Smoking was also associated with increased overall mortality in hypertensive patients (HR=1.993; 95% CI: 1.766–2.249). Mediation analysis revealed that systemic inflammation, as measured by SII, accounted for 87.70% of the increased mortality in hypertensive smokers (ACME=0.068, p<0.001).

**CONCLUSIONS:**

This study establishes a significant association between smoking, HTN and mortality. The findings underscore a potential dose-response trend between cigarette consumption and HTN, with systemic inflammation playing a key role in mediating the higher mortality observed in hypertensive smokers. Interventions targeting smoking cessation and systemic inflammation may significantly reduce the burden of HTN-related morbidity and mortality.

## INTRODUCTION

Cigarette smoking remains a leading global cause of preventable morbidity and mortality, posing a substantial public health burden^1^. It is a well-established major risk factor for a spectrum of cardiovascular diseases (CVDs), including atherosclerosis, coronary heart disease, and stroke^2^. Despite its established role in broader cardiovascular health, the specific association between smoking and hypertension (HTN) remains inconsistent across the literature^3-6^. These discrepancies are particularly evident when examining different smoking statuses. For current smokers, the evidence is mixed: several cross-sectional studies have reported a significant positive association with HTN^7,8^, while others, including some longitudinal investigations, have found no significant link or even an inverse relationship^6,7^. Similarly, studies on former smokers are divided, with some indicating a significantly elevated likelihood of HTN compared to never or current smokers^6,7^, while others do not support this finding. The heterogeneity in these results is likely multifactorial, arising from variations in study design, population characteristics, and adjustments for key confounders such as BMI and socioeconomic status (SES)^9,10^. Collectively, these inconsistencies underscore the critical need for more rigorous, large-scale studies to clarify the relationship between smoking and HTN.

The heterogeneity in smoking behavior itself – encompassing smoking status, intensity (cigarettes per day), and duration – may be a critical source of these inconsistent findings across studies. For instance, an analysis of the UK Biobank demonstrated that while current smokers had a lower observational likelihood of hypertension compared to never smokers, a higher smoking intensity was paradoxically associated with an increased risk^11^. This highlights that different aspects of smoking may have distinct and even opposing effects on blood pressure, and a nuanced investigation that considers both smoking status and volume is warranted.

Beyond observational associations, the question of causality between smoking and HTN remains open. Mendelian randomization (MR) studies, which use genetic variants as instrumental variables to minimize confounding, have provided mixed evidence. A recent review of the observational and genetic evidence concluded that while observational data often show a complex, sometimes paradoxical relationship, MR analyses do not consistently support a causal relationship between smoking behavior and HTN^11^. These findings suggest that the observational associations may be influenced by residual confounding or other biases. Nevertheless, elucidating the potential biological pathways, such as systemic inflammation, through which smoking might influence outcomes in hypertensive patients remains a crucial endeavor for risk stratification and intervention, regardless of the ultimate causal nature of the association.

In this context, quantifying systemic inflammation is key. The systemic inflammation index (SII) – a novel and integrated hematologic biomarker calculated as: (neutrophil count × platelet count)/lymphocyte count, has emerged as a powerful, objective measure of the body’s inflammatory status^12,13^. SII has demonstrated superior prognostic value over individual cell counts in predicting mortality across various conditions, including cardiovascular diseases, cancers, and diabetes^14-19^. Critically, smoking is a known potent driver of chronic inflammation^20,21^, which is also a pathophysiological hallmark of hypertension. However, the interplay between smoking, SII, and mortality specifically within the hypertensive population remains uninvestigated. It is unknown whether SII serves as a key mechanistic link explaining the heightened mortality risk observed among hypertensive smokers.

Beyond its debated relationship with HTN, smoking exerts a profound and unequivocal impact on overall mortality. It is well-established that current smokers face a mortality rate more than three times higher than never smokers^22^. Importantly, the benefits of smoking cessation are clear, with studies showing that former smokers, including older adults, have a substantially reduced risk of all-cause mortality compared to current smokers, and this risk declines further the longer cessation is maintained^23,24^. Smoking is significantly associated with increased mortality from cardiovascular diseases (CVDs), including ischemic heart disease and stroke^22^, as well as numerous other conditions^22,25,26^. Despite this robust evidence base, there is a critical gap in the literature: no studies have specifically investigated the impact of smoking on mortality rates in the large and vulnerable population of patients with established HTN.

To address these gaps in the literature, the present study utilized data from ten cycles of the National Health and Nutrition Examination Survey (NHANES) from 1999 to 2018. This study aimed to: 1) investigate the association between smoking and HTN, comprehensively examining both smoking status and smoking volume; 2) assess the impact of smoking on all-cause mortality specifically within the hypertensive population; and 3) explore the potential mediating role of systemic inflammation, as measured by the systemic inflammation index (SII), in the relationship between smoking and mortality among hypertensive patients.

## METHODS

### Study population

**Figure 1:**
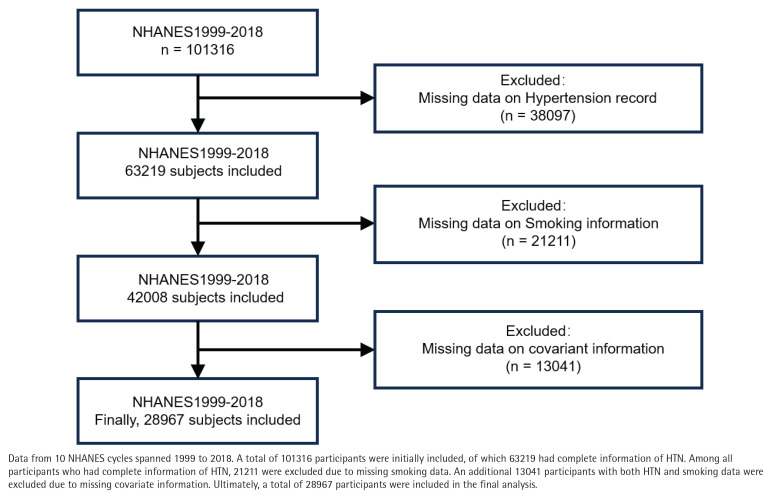
Flowchart of participant selection in the cross-sectional analysis of the US population from NHANES 1999–2018

NHANES is a complex, multistage, nationally representative, standardized, and stratified cross-sectional study that focuses on health and nutrition risk factors in the US population. NHANES has been conducted biennially since 1999, with a different cohort of participants in each cycle. Data collection involves home interviews and evaluations at mobile medical examination centers^27^. We conducted a pooled secondary data analysis of 10 consecutive NHANES cycles (1999–2018), selected to avoid potential confounding from the COVID-19 pandemic that began after 2018. Both male and female respondents aged ≥20 years were eligible. We sequentially excluded 1728 participants with missing hypertension status, 1094 with incomplete smoking information, and 2436 lacking covariate data (age, sex, BMI, SES, etc.), leaving 41372 participants for analysis (Figure 1). The NHANES protocol was approved by the NCHS Research Ethics Review Board, and all participants provided written informed consent.

### Exposure: smoking

The assessment of smoking, the primary exposure variable in this study, was based on data collected through standardized NHANES questionnaires. Smoking status was determined by the question: ‘Have you ever smoked at least 100 cigarettes in your life?’. Participants who responded ‘no’ were categorized as ‘non-smokers’. Those who answered ‘yes’ were classified as smokers and were further asked about their average daily cigarette consumption over the past 30 days, which we defined as ‘smoking volume’. Smoking volume was categorized into six groups: 0 (non-smokers), 1–5 (Q1), 6–10 (Q2), 11–20 (Q3), 21–30 (Q4), and >30 cigarettes/day (Q5)^28^. This classification allowed for a detailed exploration of the quantitative relationship between smoking volume and HTN, as well as the associated mortality among hypertensive patients.

### Outcome: HTN of hypertensive patients

HTN case status was ascertained through a combination of physical examination data and self-reported information from the NHANES interview components. Following the NHANES Physician Examination Protocol, certified examiners measured blood pressure using a standard mercury sphygmomanometer. Participants were required to have rested quietly in a seated position for 5 minutes prior to measurement. Three to four consecutive readings were recorded on the participant’s right arm. The mean of all but the first reading was calculated to define the average SBP and DBP. Measured HTN was defined as an average SBP ≥140 mmHg or DBP ≥90 mmHg^17^. Self-reported information on hypertension diagnosis and medication use was collected from the Medical Conditions Questionnaire (MCQ). Specifically, a positive history of diagnosed HTN was defined by a ‘yes’ response to variable MCQ080 (‘Has a doctor or other health professional ever told you that you have hypertension?’). Current use of antihypertensive medication was defined by a ‘yes’ response to variable MCQ090G (‘Because of your high blood pressure/hypertension, are you now taking prescribed medicine?’). A participant was defined as having HTN if they met any of the above three criteria.

### Mediated variables: systemic inflammation index (SII)

The systemic inflammation index (SII) was calculated for each participant as a key marker of systemic inflammation. The required hematologic parameters (neutrophil, lymphocyte, and platelet counts) were obtained from the NHANES complete blood count (CBC) data. These blood samples were collected by trained phlebotomists during the mobile examination center visit and analyzed in certified laboratories using automated hematology analyzers. The SII was computed using the formula: SII (×10^9^/L)=(neutrophil count ×10^9^/L) × (platelet count ×10^9^/L)/(lymphocyte count ×10^9^/L)^12^. SII was selected as the primary inflammatory marker because it is a composite index derived from routine and reliably measured CBC parameters, and it has been extensively validated in the literature as a robust prognostic indicator for cardiovascular and metabolic diseases^18,29^.

### Survival data of hypertensive patients

Endpoints and follow-up data for hypertensive patients were obtained by linking participant records to the National Death Index public access files. Mortality status was determined using the ‘MORTSTAT’ variable, and the ‘PERMTH_EXM’ variable provided the follow-up time in months.

For the specific purpose of conducting the mediation analysis, hypertensive participants were categorized into two distinct groups based on their survival outcome and follow-up time, a method adapted from prior studies^19,30^. The low mortality group was defined as participants who were alive at the end of follow-up and had a survival time greater than the 75th percentile of the overall cohort. Conversely, the high mortality group was defined as participants who had died with a survival time less than the 25th percentile.

### Covariates

Covariates were selected based on prior knowledge and their potential confounding role. Data on sociodemographic factors, including age (treated as continuous), gender (male/female), race/ethnicity (categorized as Mexican American, Other Hispanic, Non-Hispanic White, Non-Hispanic Black, and Other Race), education level (categorized as lower than grade 9, 9–11 grade, high school graduate/GED, some college or AA degree, and college graduate or higher), marital status (married, widowed, divorced, separated, never married, living with partner), and the family poverty-to-income ratio (PIR, treated as continuous), were collected through standardized household interviews. Body mass index (BMI, kg/m^2^) was calculated from objectively measured weight and height obtained during the physical examination. Cardiovascular conditions were identified based on self-reported physician diagnoses from the Medical Conditions Questionnaire. Specifically, we included heart failure, coronary heart disease (CHD), angina (also known as angina pectoris), heart attack (myocardial infarction), and stroke. Diabetes mellitus was defined as meeting any of the following criteria: 1) hemoglobin AIC >6.5%; 2) fasting plasma glucose ≥126 mg/dL; 3) self-reported current use of antidiabetic medications; and 3) a self-reported physician diagnosis of diabetes^31^.

### Statistical analysis

All analyses were performed using R software (version 4.3.2). Continuous variables included age, BMI, family PIR, and SII, while all other variables (e.g. gender, race, education level, smoking status/volume) were treated as categorical. Continuous variables are presented as means ± standard errors in the mean, and categorical variables as frequencies and percentages. Group differences were assessed using ANOVA/Kruskal-Wallis tests for continuous variables and chi-squared tests for categorical variables.

The associations between smoking status, smoking volume, and HTN were examined using three logistic regression models. Model fit was assessed using pseudo-R^2^ (McFadden’s, Nagelkerke’s, Cox & Snell’s), Hosmer-Lemeshow test, AIC, and BIC.

To minimize confounding, we performed 1:1 propensity score matching (PSM) without replacement using a caliper of 0.2 SD of the propensity score logit. The propensity model included age, gender, BMI, race, education level, marital status, family PIR, alcohol use, and diabetes. Balance was evaluated with absolute standardized mean differences (ASMD <0.1 indicated good balance).

In the matched dataset, the dose-response relationship between smoking volume and HTN was modeled using restricted cubic splines (RCS) within logistic regression. The model used 4 knots (at the 5th, 35th, 65th, and 95th percentiles), with the 5th percentile as the reference.

Among hypertensive patients, the association between smoking and all-cause mortality was analyzed. Follow-up time was derived from the ‘PERMTH_EXM’ variable. Survival curves were generated using the Kaplan-Meier method and compared with the log-rank test. Hazard ratios were estimated from Cox proportional hazards models, adjusted for age, gender, BMI, race, education level, marital status, family PIR, alcohol use, diabetes, and cardiovascular conditions (heart failure, CHD, angina, heart attack, stroke). The proportional hazards assumption was verified using Schoenfeld residuals (global p>0.05).

Causal mediation analyses were performed to quantify the proportion of the total effect of smoking on high mortality that was mediated through various clinical pathways. The primary mediator of interest was the systemic inflammation index (SII). Additionally, to provide a comprehensive assessment and contextualize the role of systemic inflammation, we explored the mediating effects of several other smoking-related clinical conditions and biomarkers, including: systolic hypertension (SH), stroke, coronary heart disease (CHD), heart attack, heart failure, angina, diabetes, and body mass index (BMI). For each candidate mediator, we fitted a mediator model (mediator ~ smoking + covariates) and an outcome model (mortality ~ smoking + mediator + covariates). The average causal mediation effect (ACME), average direct effect (ADE), and the proportion mediated were estimated for each pathway via quasi-Bayesian Monte Carlo simulation with 1000 iterations.A two-sided p<0.05 defined statistical significance.

## RESULTS

### Smoking increases the likelihood of HTN

Data from 10 NHANES cycles between 1999 and 2018 were analyzed, comprising 101316 participants. After excluding 13041 participants due to missing covariate data, the final analysis included 28967 participants, representing the largest retrospective clinical study to date examining the relationship between smoking and HTN (Figure 1).

Baseline characteristics of patients with or without HTN are shown in Table 1. The study population consisted of 13055 males and 15902 females. Approximately 28.1% (8126 participants) were diagnosed with HTN (p=0.42 for sex difference). Participants with HTN were more likely to be older, married, non-Hispanic White females, have a higher level of education, lower family PIR, and higher BMI. Among participants with normal blood pressure, 71.5% (n=14252) were non-smokers and 28.5% (n=5690) were smokers. Conversely, among participants with HTN, 27.0% (n=2436) were non-smokers, while 73.0% (n=6579) were smokers. HTN participants were also more likely to consume alcohol and less likely to have diabetes. Cardiovascular diseases (CVDs), including heart failure, coronary heart disease (CHD), angina, heart attack, and stroke, were relatively uncommon in the population but occurred significantly more frequently in individuals with HTN.

**Table 1:** Baseline characteristics of participants with and without hypertension in a cross-sectional analysis of US adults, NHANES 1999–2018 (N=28967)

*Characteristics*	*Categories*	*Overall (N=28957)* *n (%)*	*Non-HTN (N=19942)* *n (%)*	*HTN (N=9015)* *n (%)*	*p*
**Smoking status**	Non-smoker	20831 (71.9)	14252 (71.5)	6579 (73.0)	0.008
	Smoker	8126 (28.1)	5690 (28.5)	2436 (27.0)	
**Smoking volume** (cigarettes/day)	Non-smoker	20831 (71.9)	14252 (71.5)	6579 (73.0)	0.002
	Q1 (1–5)	2395 (8.3)	1714 (8.6)	681 (7.6)	
	Q2 (6–10)	2206 (7.6)	1569 (7.9)	637 (7.1)	
	Q3 (11–20)	2672 (9.2)	1833 (9.2)	839 (9.3)	
	Q4 (21–30)	515 (1.8)	358 (1.8)	157 (1.7)	
	Q5 (>30)	338 (1.2)	216 (1.1)	122 (1.4)	
**Age** (years), mean (SEM)		46.60 (0.1)	41.61 (0.11)	57.62 (0.16)	<0.001
**Gender**	Male	13055 (45.1)	9217 (46.2)	3838 (42.6)	<0.001
	Female	15902 (54.9)	10725 (53.8)	5177 (57.4)	
**Race**	Mexican American	5061 (17.5)	3848 (19.3)	1213 (13.5)	<0.001
	Other Hispanic	2310 (8.0)	1634 (8.2)	676 (7.5)	
	Non-Hispanic White	12704 (43.9)	8726 (43.8)	3978 (44.1)	
	Non-Hispanic Black	6263 (21.6)	3765 (18.9)	2498 (27.7)	
	Other Race	2619 (9.0)	1969 (9.9)	650 (7.2)	
**Education level**	Lower than grade 9	2964 (10.2)	1840 (9.2)	1124 (12.5)	<0.001
	9–11 grade	4231 (14.6)	2821 (14.1)	1410 (15.6)	
	Highschool graduate	6741 (23.3)	4491 (22.5)	2250 (25.0)	
	Some college or AA degree	8455 (29.2)	5869 (29.4)	2586 (28.7)	
	College graduate or higher	6566 (22.7)	4921 (24.7)	1645 (18.2)	
**Marital status**	Married	14755 (51.0)	10100 (50.6)	4655 (51.6)	<0.001
	Widowed	2103 (7.3)	821 (4.1)	1282 (14.2)	
	Divorced	2972 (10.3)	1780 (8.9)	1192 (13.2)	
	Separated	1001 (3.5)	628 (3.1)	373 (4.1)	
	Never married	5736 (19.8)	4735 (23.7)	1001 (11.1)	
	Living with partner	2390 (8.3)	1878 (9.4)	512 (5.7)	
**Family PIR**, mean (SEM)		2.51 (0.01 )	2.54 (0.01)	2.44 (0.02)	<0.001
**BMI** (kg/m^2^), mean (SEM)		28.85 (0.04 )	27.86 (0.05)	31.03 (0.08)	<0.001
**Alcohol**	Yes	19708 (68.1)	14093 (0.05)	5615 (0.08)	<0.001
	No	9249 (31.9)	5849 (29.3)	3400 (37.7)	
**Diabetes**	Yes	3895 (13.5)	1428 (7.2)	2467 (27.4)	<0.001
	No	25062 (86.5)	18514 (92.8)	6548 (72.6)	
**Heart failure**	No	28270 (97.6)	19785 (99.2)	8485 (94.1)	<0.001
	Yes	687 (2.4)	157 (0.8)	530 (5.9)	
**CHD**	No	28133 (97.2)	19731 (98.9)	8402 (93.2)	<0.001
	Yes	824 (2.8)	211 (1.1)	613 (6.8)	
**Angina**	No	28317 (97.8)	19778 (99.2)	8539 (94.7)	<0.001
	Yes	640 (2.2)	164 (0.8)	476 (5.3)	
**Heart attack**	No	28047 (96.9)	19678 (98.7)	8369 (92.8)	<0.001
	Yes	910 (3.1)	264 (1.3)	646 (7.2)	
**Stroke**	No	28097 (97.0)	19712 (98.8)	8385 (93.0)	<0.001
	Yes	860 (3.0)	230 (1.2)	630 (7.0)	

PIR: poverty income ratio. CHD: coronary heart disease. Continuous variables are presented as mean and standard error in the mean, and were compared using Student’s t-test. Categorical variables are presented as frequencies and percentages and were compared using the chi-squared test.

Baseline characteristics of smokers and non-smokers (Table 2) revealed significant differences in age, gender, BMI, race, education level, marital status, family PIR, and alcohol consumption (p<0.05). Smoking was strongly associated with diabetes, heart failure, angina, heart attack, and stroke (p<0.05). Smokers were more likely to be younger, male, non-Hispanic White or Black, with lower BMI, family PIR, and education level. Smokers also reported higher rates of conditions such as chronic bronchitis, emphysema, depression, and heart failure.

**Table 2:** Baseline characteristics of the study participants by smoking status (N=28967)

*Characteristics*	*Categories*	*Overall* *(N=28957)* *n (%)*	*Non-smoker* *(N=20831)* *n (%)*	*Smoker* *(N=8126)* *n (%)*	*p*
**HTN**	No	19942 (68.9)	14252 (68.4)	5690 (70.0)	0.008
	Yes	9015 (31.1)	6579 (31.6)	2436 (30.0)	
**Age** (years), mean (SEM)		46.60 (0.1)	47.48 (0.13)	44.32 (0.17)	<0.001
**Gender**	Male	13055 (45.1)	8422 (40.4)	4633 (57.0)	<0.001
	Female	15902 (54.9)	12409 (59.6)	3493 (43.0)	
**Race**	Mexican American	5061 (17.5)	4020 (19.3)	1041 (12.8)	<0.001
	Other Hispanic	2310 (8.0)	1815 (8.7)	495 (6.1)	
	Non-Hispanic White	12704 (43.9)	8556 (41.1)	4148 (51.0)	
	Non-Hispanic Black	6263 (21.6)	4343 (20.8)	1920 (23.6)	
	Other Race	2619 (9.0)	2097 (10.1)	522 (6.4)	
**Education level**	Lower than grade 9	2964 (10.2)	2225 (10.7)	739 (9.1)	<0.001
	9–11 grade	4231 (14.6)	2386 (11.5)	1845 (22.7)	
	Highschool graduate	6741 (23.3)	4305 (20.7)	2436 (30.0)	
	Some college or AA degree	8455 (29.2)	6087 (29.2)	2368 (29.1)	
	College graduate or higher	6566 (22.7)	5828 (28.0)	738 (9.1)	
**Marital**	Married	14755 (51.0)	11581 (55.6)	3174 (39.1)	<0.001
	Widowed	2103 (7.3)	1669 (8.0)	434 (5.3)	
	Divorced	2972 (10.3)	1753 (8.4)	1219 (15.0)	
	Separated	1001 (3.5)	587 (2.8)	414 (5.1)	
	Never married	5736 (19.8)	3922 (18.8)	1814 (22.3)	
	Living with partner	2390 (8.3)	1319 (6.3)	1071 (13.2)	
**Family PIR**, mean (SEM)		2.51 (0.01)	2.71 (0.01)	2.02 (0.02)	<0.001
**BMI** (kg/m^2^), mean (SEM)		28.85 (0.04 )	29.17 (0.06)	28.03 (0.07)	<0.001
**Alcohol**	Yes	19708 (68.1)	12776 (61.3)	6932 (85.3)	<0.001
	No	9249 (31.9)	8055 (38.7)	1194 (14.7)	
**Diabetes**	Yes	3895 (13.5)	2941 (14.1)	954 (11.7)	<0.001
	No	25062 (86.5)	17890 (85.9)	7172 (88.3)	
**Heart failure**	No	28270 (97.6)	20375 (97.8)	7895 (97.2)	0.001
	Yes	687 (2.4)	456 (2.2)	231 (2.8)	
**CHD**	No	28133 (97.2)	20261 (97.3)	7872 (96.9)	0.08
	Yes	824 (2.8)	570 (2.7)	254 (3.1)	
**Angina**	No	28317 (97.8)	20405 (98.0)	7912 (97.4)	0.003
	Yes	640 (2.2)	426 (2.0)	214 (2.6)	
**Heart attack**	No	28047 (96.9)	20295 (97.4)	7752 (95.4)	<0.001
	Yes	910 (3.1)	536 (2.6)	374 (4.6)	
**Stroke**	No	28097 (97.0)	20289 (97.4)	7808 (96.1)	<0.001
	Yes	860 (3.0)	542 (2.6)	318 (3.9)	

HTN:hypertension. PIR: poverty income ratio. CHD: coronary heart disease. Continuous variables are presented as mean and standard error in the mean, and were compared using Student’s t-test. Categorical variables are presented as frequencies and percentages and were compared using the chi-squared test.

### Potential confounders and mediators

**Figure 2:**
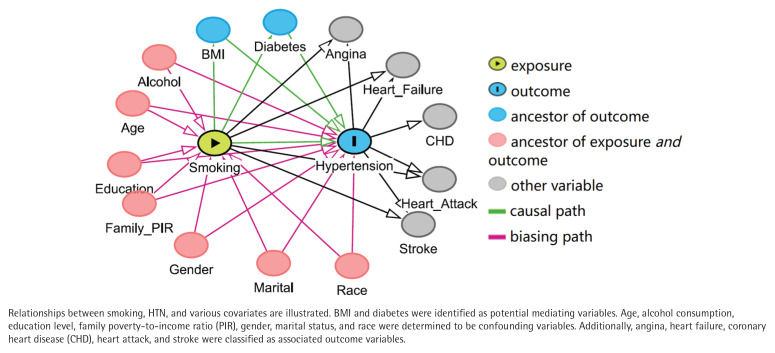
Directed acyclic graph of the presumed relationships among smoking, hypertension, and covariates (N=28967)

Directed acyclic graphs (Figure 2) demonstrated that BMI and diabetes may serve as intermediate variables, while age, alcohol consumption, education level, family PIR, gender, marital status, and race were identified as potential confounding variables. CVDs were classified as related outcomes.

Multivariate analyses (Table 3) showed that smoking significantly increased the odds of HTN in all three models. Compared to non-smokers, smokers had a 1.32-fold increased likelihood of HTN (95% CI: 1.24–1.41, p<0.001) after adjusting for age, gender, and BMI, and a 1.18-fold increased likelihood (95% CI: 1.10–1.27, p<0.001) after adjusting for all confounders, including education level, marital status, family PIR, diabetes, and alcohol use.

**Table 3:** Odds ratios for hypertension by smoking status and volume (N=28967)

*Variables*	*Model 1*			*Model 2*			*Model 3*		
	*OR*	*95% CI*	*p*	*AOR*	*95% CI*	*p*	*AOR*	*95% CI*	*p*
**Smoking status**	0.93	0.88–0.98	0.008	1.32	1.24–1.41	0.001	1.18	1.10–1.27	0.001
**Smoking volume** (cigarettes/day)									
Non-smoker									
Q1 (1–5)	0.86	0.78–0.95	0.002	1.32	1.19–1.46	<0.001	1.21	1.09–1.35	<0.001
Q2 (6–10)	0.88	0.80–0.97	0.010	1.31	1.17–1.45	<0.001	1.11	0.99–1.24	0.0596
Q3 (11–20)	0.99	0.91–1.08	0.848	1.34	1.22–1.48	<0.001	1.21	1.08–1.34	<0.001
Q4 (21–30)	0.95	0.79–1.15	0.597	1.17	0.95–1.44	0.135	1.11	0.90–1.37	0.341
Q5 (>30)	1.22	0.98–1.53	0.077	1.51	1.18–1.92	<0.001	1.37	1.07–1.75	0.013

Model 1: unadjusted. AOR: adjusted odds ratio. Model 2: adjusted for age, gender, and BMI. Model 3: adjusted as for Model 2 plus race, education level, marital status, family PIR, diabetes and alcohol use. Model fit indices for fully adjusted models (Model 3): Model 3a (Smoking status): AIC=28206.37, Nagelkerke’s R^2^=0.330, Hosmer-Lemeshow test: χ^2^=39.92, p<0.001; Model 3b (Smoking volume): AIC=28211, Nagelkerke’s R^2^=0.331, Hosmer-Lemeshow test: χ^2^=39.15, p <0.001.

### Cigarette consumption positively correlates with the likelihood of HTN

Participants in the Q5 (>30 cigarettes/day) group showed significantly higher likelihood of HTN (p=0.002) compared to the other groups before adjustment (Table 3, Model 1). Adjusted analyses (Table 3, Model 3) further confirmed a dose-response relationship between cigarette consumption and HTN. The adjusted odds ratio (AOR) of HTN was 1.37 (95% CI: 1.07–1.75; p=0.013) for participants who smoked more than 30 cigarettes/day compared to non-smokers. While no significant association with HTN was found for the Q2 (6–10 cigarettes/day) or Q4 (21–30 cigarettes/day) groups, the likelihood of HTN was significantly increased in the Q1 (AOR=1.21; 95% CI: 1.09–1.35) and Q3 (AOR=1.21; 95% CI: 1.08–1.34) groups compared to non-smokers.

### PSM analysis confirms smoking increases the likelihood of HTN

**Figure 3:**
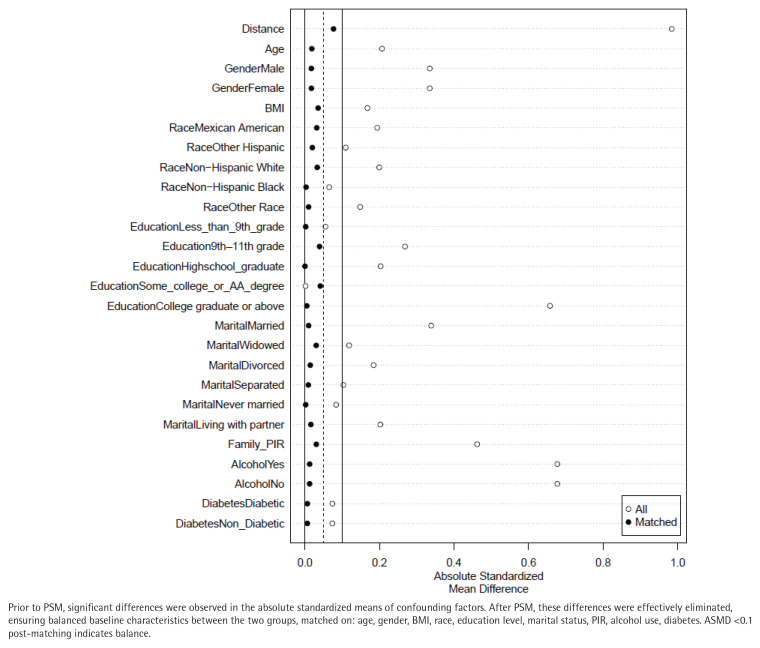
Balance of covariates before and after propensity score matching

PSM was conducted to control for confounding factors, including age, gender, BMI, race, education level, marital status, family PIR, alcohol consumption, and diabetes. Before PSM, 28967 participants were included; after PSM, 14586 matched participants remained. Post-PSM analysis eliminated differences in baseline covariates between smokers and non-smokers (Figure 3; and Supplementary file Figure S1).

**Figure 4:**
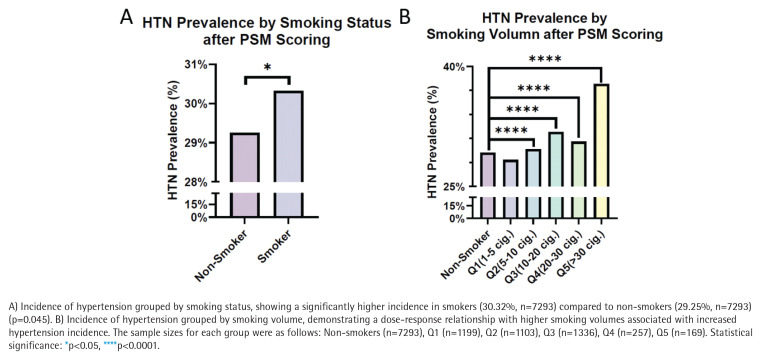
Incidence of hypertension by (A) smoking status and (B) smoking volume after propensity score matching (N=14586)

Statistical analysis of the matched dataset revealed that 30.32% of smokers had HTN compared to 29.25% of non-smokers, demonstrating a significant increase in the likelihood of HTN among smokers (p=0.045) (Figure 4A). Further stratification by smoking volume showed a significant increase in HTN incidence among smokers, with the highest prevalence (37.81%) in participants smoking >30 cigarettes/day (p<0.0001) (Figure 4B).

### Dose-response relationship between smoking volume and HTN in the entire population

To complement the PSM analysis and fully utilize the available data, we further employed a restricted cubic spline (RCS) model to examine the continuous dose-response relationship between smoking volume and HTN in the full population of smokers (n=8126), with adjustment for age, gender, BMI, race, education level, marital status, family PIR, alcohol use, and diabetes.

**Figure 5:**
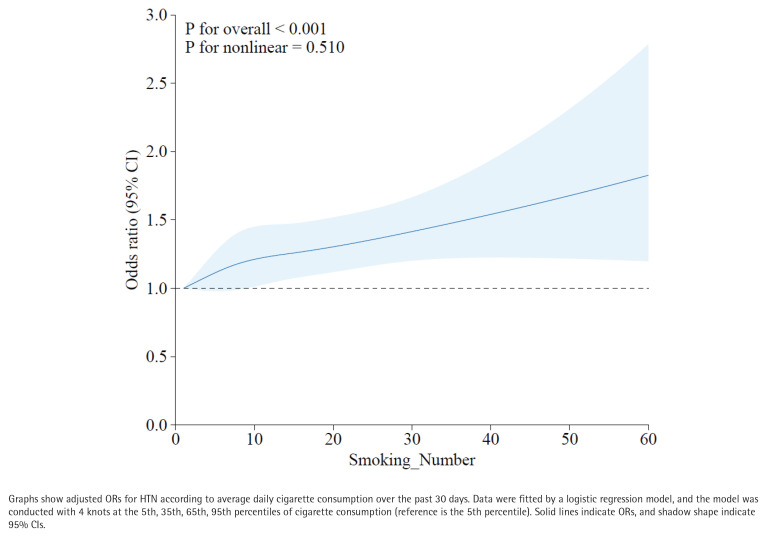
Dose-response relationship between smoking volume and hypertension modeled using restricted cubic splines (N=14586)

This analysis revealed a significant overall association (p for overall <0.001) and a linear increase in the odds of HTN with rising cigarette consumption (p for nonlinear = 0.510) (Figure 5). The curve demonstrates that the likelihood of HTN increases steadily with the number of cigarettes smoked per day, reinforcing the dose-response relationship observed in the categorical analysis.

### Smoking increases overall mortality in hypertensive patients

Smoking was significantly associated with increased all-cause mortality compared to non-smokers (p<0.001) (Supplementary file Figure 1A). Stratifying hypertensive patients by smoking volume revealed a positive correlation between higher cigarette consumption and all-cause mortality (p<0.001), with participants smoking >30 cigarettes/day exhibiting the highest mortality (Figure 4B). After adjusting for confounders, the hazard ratio (HR) for all-cause mortality among smokers was 1.99 (95% CI: 1.77–2.25, p<0.001) compared with non-smokers (Supplementary file Figure 2).

### Systemic inflammation mediates mortality in hypertensive smokers

To investigate the mechanism underlying the likelihood of increased mortality in hypertensive smokers, systemic inflammation index (SII) was analyzed and was found to be significantly higher in smokers compared to non-smokers (mean ± SE: 1081 ± 35.4 vs 795 ± 42.1; p<0.001) and in the high mortality group compared to the low mortality group (mean ± SE: 1119 ± 36.9 vs 387 ± 12.8; p<0.001) (Supplementary file Figure 3).

We conducted a series of mediation analyses to explore the extent to which various clinical conditions and biomarkers mediated the relationship between smoking and higher mortality in hypertensive patients. The results of these analyses are summarized in Supplementary file Figure 4. Among all the mediators examined, the systemic inflammation index (SII) demonstrated the most substantial and significant mediating effect.

Mediation analysis revealed that SII mediated 87.70% (95% CI: 50.20–193, p<0.001) of the causal relationship between smoking and increased mortality in hypertensive patients. The average causal mediation effect (ACME) was 0.068 (95% CI: 0.043–0.09, p<0.001), while the average direct effect (ADE) was not significant (0.013; 95% CI: -0.031–0.06, p=0.80).

The mediation analysis revealed that SII accounted for 87.70% of the total effect, while the average direct effect (ADE) of smoking on mortality, independent of SII, was not statistically significant. For the non-specialist, this statistical pattern – a large, significant indirect effect through the mediator coupled with a non-significant direct effect – is highly suggestive of a model in which systemic inflammation serves as the predominant pathway linking smoking to higher mortality in this patient population. It indicates that the harmful impact of smoking on survival in hypertensive patients is largely, if not entirely, explained by its propensity to drive systemic inflammation. However, in the context of an observational study, we cannot definitively rule out other minor or unmeasured direct pathways.

## DISCUSSION

### Restatement of aims and main findings

This study sought to clarify the relationship between smoking and hypertension (HTN) and to explore the underlying mechanism linking smoking to mortality in hypertensive patients. Utilizing a large, nationally representative sample from NHANES, our analysis yielded three principal findings. First, we observed a significant positive association between smoking and HTN, which exhibited a clear potential dose-response relationship with cigarette consumption. Second, among individuals with established HTN, smokers had substantially higher all-cause mortality than non-smokers. Third, mediation analysis indicated that systemic inflammation, quantified by the systemic inflammation index (SII), acted as a significant mediator, accounting for a large proportion (87.70%) of the association between smoking and elevated mortality in this population.

### Smoking-HTN association in context of existing literature

Our finding of a positive association between smoking and HTN aligns with several prior observational studies^3,8,32^. More importantly, the identified potential dose-response relationship, where higher daily cigarette consumption was linked to greater odds of HTN, is consistent with the observational findings of Jareebi et al.^11^ who also reported a modest increase in the likelihood of HTN per additional cigarette smoked per day. This consistency strengthens the evidence for a potential dose-response relationship between smoking intensity and HTN.

However, the broader literature remains conflicting, with some studies reporting null or even inverse associations^6,33^. These discrepancies may be attributed to variations in study populations, adjustments for different confounding factors, or, as suggested by Jareebi et al.^11^, the complex interplay of different smoking characteristics (e.g. status, intensity, duration) which may exert divergent effects. It is also crucial to acknowledge that Mendelian randomization studies have not consistently supported a causal relationship between smoking and HTN^11^, implying that the observational associations we and others report may be susceptible to residual confounding from unmeasured lifestyle factors.

Our finding of a clear dose-response relationship contrasts with some studies that reported no association between smoking intensity and continuous blood pressure measures^34,35^. This discrepancy may be explained by key methodological differences. Firstly, the outcomes differ: we assessed clinical HTN (a diagnostic threshold), whereas null studies often analyzed continuous blood pressure values. Smoking may have a more pronounced effect on crossing a clinical disease threshold than on shifting population-wide BP levels. Secondly, the meta-analysis finding no causal association^35^ used genetic instruments, which may not capture the same exposure as our direct observational approach. Finally, our large sample size and precise smoking quantification likely enhanced our power to detect this gradient.

### Mortality findings among hypertensive smokers

While the elevated overall mortality among smokers is well-documented^36^, our study provides specific evidence for the hypertensive population. We found that all-cause mortality was nearly twice as high among hypertensive patients who smoked, compared to their non-smoking counterparts. This likelihood escalated sharply with increasing cigarette consumption, highlighting a grave concern for heavy smokers with HTN. To our knowledge, this is one of the first studies to delineate this relationship and its dose-dependent nature specifically within a nationally representative cross-sectional sample of hypertensive individuals, underscoring the critical importance of smoking cessation as part of HTN management.

### SII as a potential mechanistic pathway

Smoking is a known driver of chronic inflammation^20,21^. The systemic inflammation index (SII) is a novel marker that integrates neutrophil, lymphocyte, and platelet counts to represent the level of systemic inflammation and has been reported to effectively predict survival outcomes in numerous diseases, including cardiovascular diseases, various cancers, and others^14,15^. However, prior to our study, the relationship between smoking and SII, and specifically whether SII mediates the increased mortality among hypertensive smokers, had not been investigated.

A novel finding of our study is the identification of systemic inflammation as a potential mechanistic pathway. We demonstrated that SII levels were significantly higher in smokers and in hypertensive patients with a likelihood of high mortality . The mediation analysis revealed that SII explained a substantial portion of the smoking-mortality link. This suggests that smoking may exacerbate mortality in hypertensive patients by amplifying systemic inflammation.

However, it is important to interpret this finding with caution. As rightly noted, the systemic inflammation index (SII) is a composite measure derived from platelet, neutrophil, and lymphocyte counts, all of which are non-specific markers that can be elevated by a wide range of acute and chronic inflammatory conditions beyond smoking, such as infections, autoimmune diseases, malignancies, and other metabolic syndromes. While our analyses adjusted for several major conditions including diabetes and cardiovascular diseases, we acknowledge that residual confounding from unmeasured or subclinical inflammatory sources cannot be fully excluded. Therefore, SII in this context may partly function as a marker of the overall inflammatory burden, which is heightened by smoking but also influenced by other factors.

### Clinical and public health implications

Our findings have direct implications for clinical practice and public health. Firstly, the observed potential dose-response relationship between smoking and HTN reinforces the necessity of routine smoking status and intensity assessment in primary care, especially for individuals at risk for or diagnosed with HTN. Secondly, the strong association between smoking and higher mortality among hypertensive patients should motivate effective, integrated intervention strategies that combine antihypertensive therapy with structured smoking cessation programs. Finally, the role of SII suggests that systemic inflammation could be a potential target for intervention. While SII itself may not be ready for routine clinical use, it underscores the value of anti-inflammatory lifestyle modifications and the need for further research into anti-inflammatory therapies in this high-risk group.

### Strengths and limitations

This study has several strengths. First, the use of a large, nationally representative sample from 10 cycles of NHANES enhances the generalizability of our findings to the non-institutionalized US adult population and provides substantial statistical power. Second, the application of rigorous methods, including propensity score matching to minimize baseline confounding and comprehensive adjustment for a wide array of covariates, strengthens the robustness of our observed associations. Finally, this is a novel study that demostrates a significant association between smoking and higher all-cause mortality specifically in hypertensive patients and identifies systemic inflammation as a key mediator of this relationship.

However, some limitations should be acknowledged. First, the cross-sectional design precludes causal inference for the association between smoking and HTN. Second, residual confounding from unmeasured or imperfectly measured lifestyle factors (e.g. diet, physical activity) may persist despite our adjustments. Third, key variables, including smoking status and SII, are subject to limitations; smoking was self-reported and SII is a non-specific inflammatory marker that can be elevated by conditions beyond smoking. Fourth, while NHANES uses a complex sampling design, the exclusion of institutionalized populations and survey non-response could introduce some selection bias.

## CONCLUSIONS

This study demonstrates that smoking is positively associated with an increased incidence of HTN, with a potential dose-response relationship. Moreover, among hypertensive patients, smoking is linked to a substantially higher all-cause mortality, a relationship that appears to be mediated in large part by smoking-induced systemic inflammation. These findings underscore the importance of smoking cessation for individuals with hypertension and provide clinical evidence that systemic inflammation may partly explain the increased mortality observed in hypertensive smokers. Future prospective studies in larger cohorts are warranted to confirm these associations and elucidate the underlying causal pathways.

## Supplementary Material



## Data Availability

The data supporting this research are available from the authors on reasonable request.
