# Supplementary material for: Smoking-driven systemic inflammation elevates mortality risk in hypertensive patients: A cross-sectional study using insights from NHANES 1999–2018
Source: Tob Induc Dis. 2026 Jan 31;24:10.18332/tid/214125. doi: 10.18332/tid/214125 (PMC12860408; doi:10.18332/tid/214125)
Supplement: Supplementary file 1 [file TID-24-13-s1.pdf]

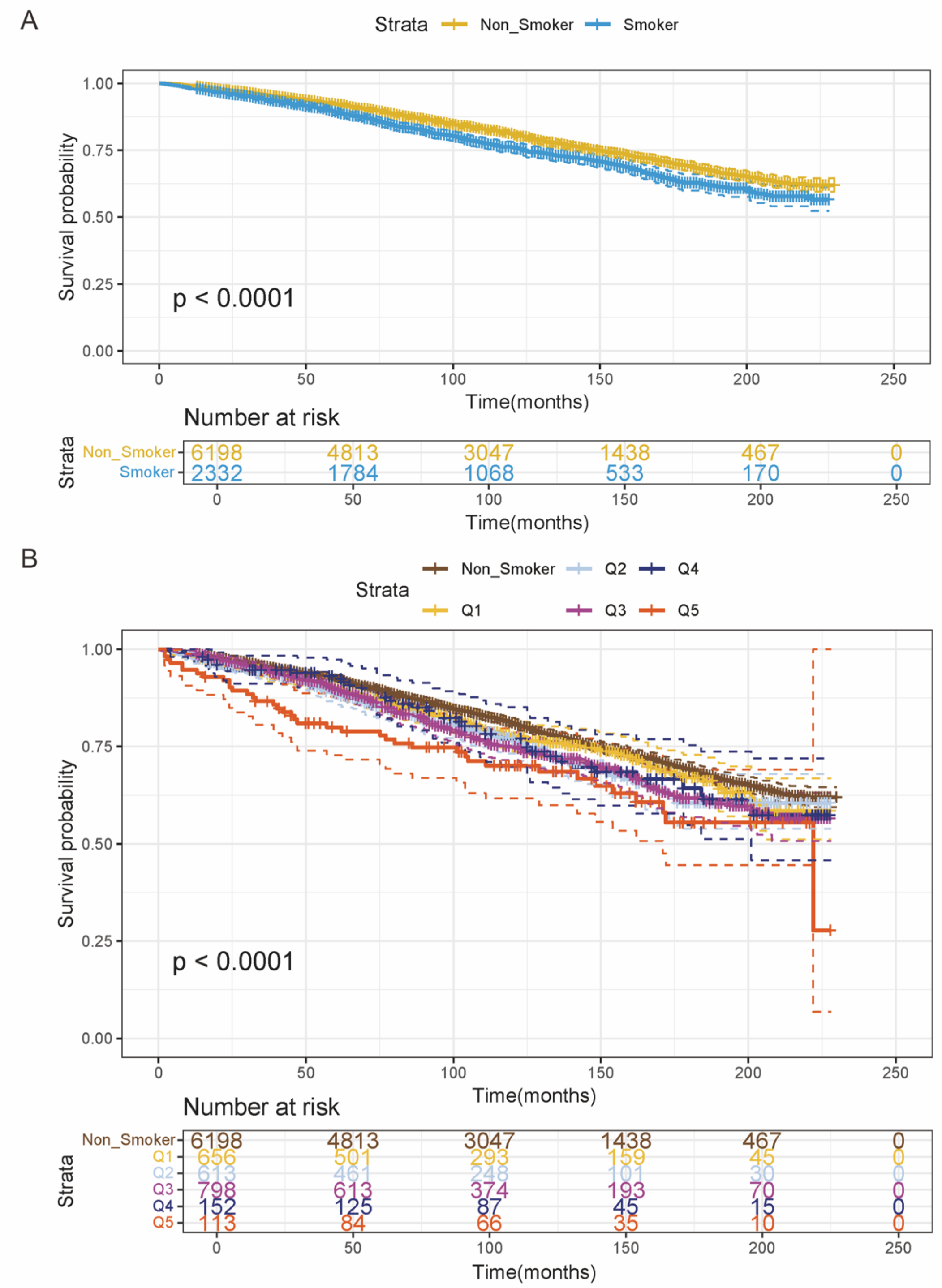

Supplementary Figure 1. Kaplan-Meier survival curves for all-cause mortality among hypertensive patients, stratified by (A) smoking status and (B) smoking volume (N = 8,530).

(A) Survival analysis between smoker and non-smoker in hypertensive patients. (B) Survival analysis grouped by stratification based on number of cigarettes smoked in patients with HTN.

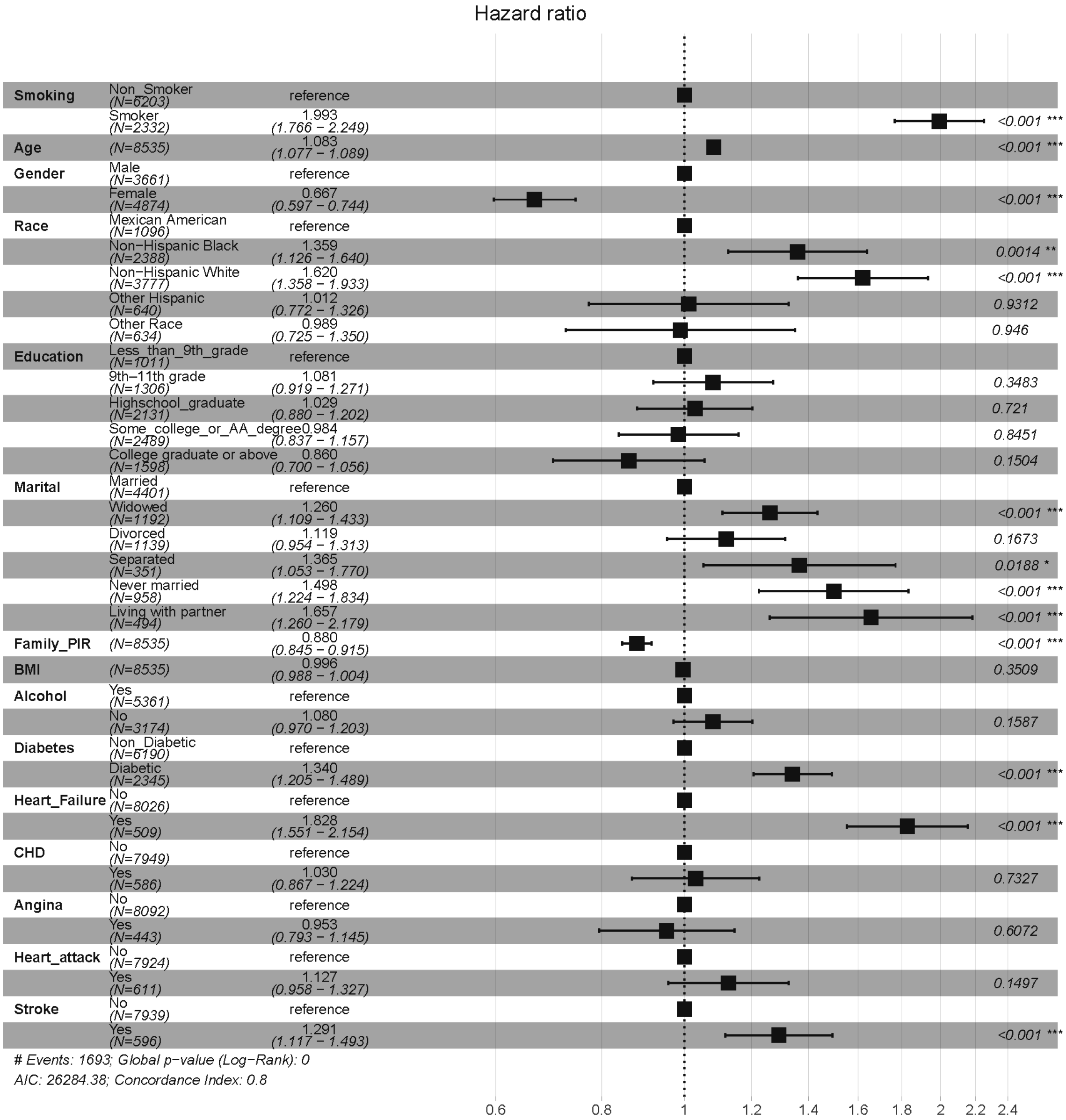

Supplementary Figure 2. Forest plot of factors associated with all-cause mortality in hypertensive patients (N = 8,530).

The Cox proportional hazards regression model included smoking status, age, gender, race, education, marital status, family poverty income ratio, BMI, alcohol use, diabetes, heart failure, coronary heart disease, angina, heart attack, and stroke. All cause mortality was the event of interest. The confidence intervals (CIs) shown are 95% CIs. Model fit indices: AIC = 26,284.38, Concordance index (C-index) = 0.80. Number of events: 1,693. Abbreviations: HR, hazard ratio; CI, confidence interval; AIC, Akaike Information Criterion; BMI, body mass index.

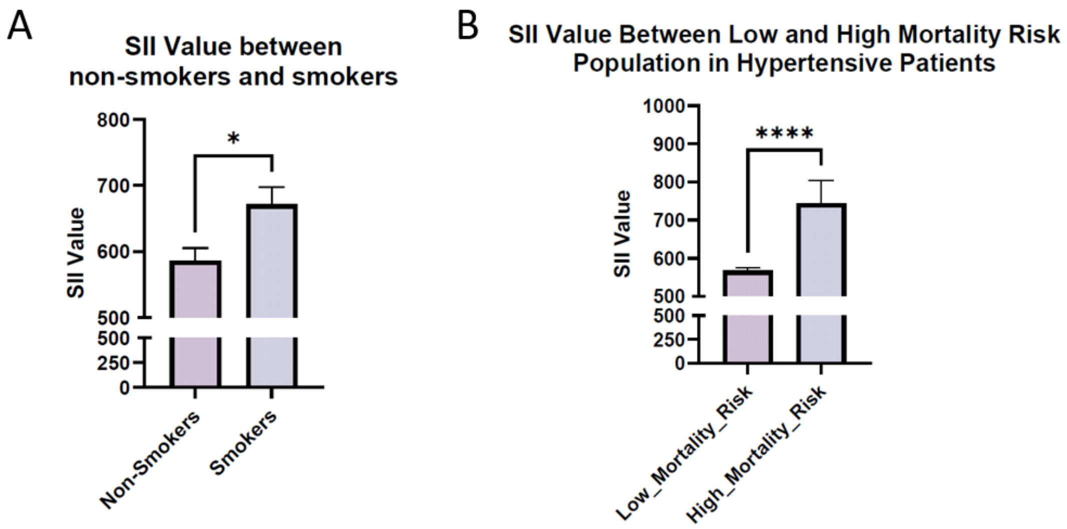

Supplementary Figure 3. Systemic Inflammation Index (SII) levels stratified by (A) smoking status and (B) mortality among hypertensive patients.

(A) Comparison of SII values between smokers (n=X1) and non-smokers (n=X2), showing significantly higher SII values in smokers. (B) Comparison of SII values between hypertensive patients in the high mortality group (n=Y1) and the low mortality group (n=Y2), indicating significantly elevated SII levels in the high mortality group. Data are presented as mean ± standard error. Between-group comparisons were performed using the Student's t-test (or Mann-Whitney U test, if applicable). Statistical significance is denoted as $*p < 0.05$ and $****p < 0.0001$.

Footnote: SII, Systemic Inflammation Index, was calculated using the formula: SII = (neutrophil count × platelet count) / lymphocyte count. All cell counts were measured in 10^9/L, and the resulting SII is a unitless value.

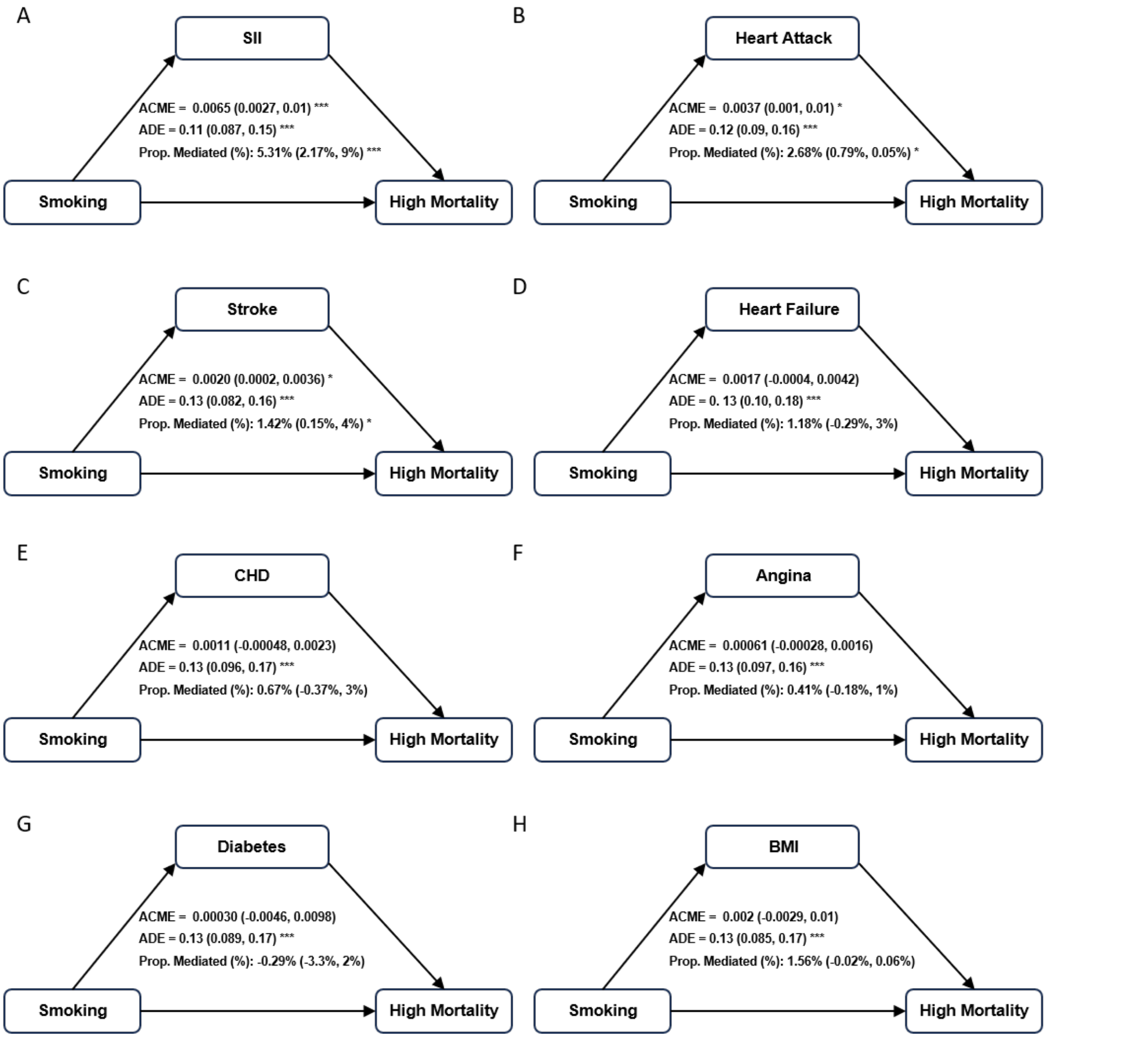

Supplementary Figure 4. Causal mediation analyses of the relationship between smoking and high mortality in hypertensive patients.

(A) Mediation analysis demonstrates that SII mediates 87.70% of the relationship between smoking and high mortality (95% CI: 50.20%–193%, $p < 0.001$). The average causal mediation effect (ACME) of SII is 0.068 (95% CI: 0.043–0.09, $p < 0.001$), while the average direct effect (ADE) is 0.013 (95% CI: -0.031–0.06, $p = 0.80$). (B-H) Results of the exploratory mediation analyses for additional factors, including heart attack, stroke, heart failure, coronary heart disease (CHD), angina, diabetes, and BMI, in the relationship between smoking and increased mortality. These analyses were performed to comprehensively assess potential mediating pathways beyond systemic inflammation. The Proportion Mediated (Prop. Mediated %) is calculated as (ACME / (ACME + ADE)) × 100%, quantifying the percentage of smoking's total effect on mortality that is explained by its ability to elevate systemic inflammation. ACME, average causal mediation effects (indirect effect). ADE, average direct effects. * p <

0.05, ** $p < 0.01$, and *** $p < 0.001$.

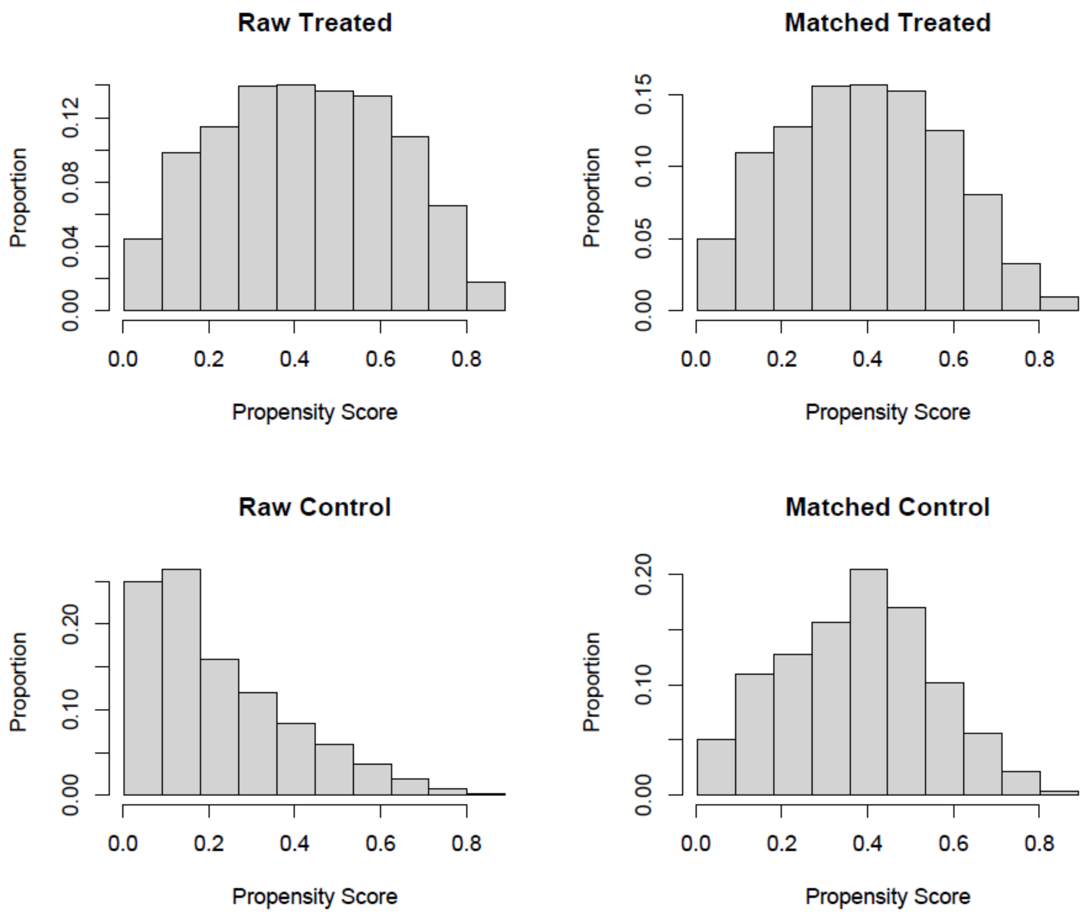

Figure S1. Elimination of Differences Between Exposed and Non-Exposed Groups After Propensity Score Matching (PSM) Analysis

Balance is shown for the exposed group (smokers) and the non-exposed group (non-smokers). PSM was performed using a 1:1 matching algorithm without replacement and a caliper width of 0.2 standard deviations of the logit of the propensity score. After matching, each group contained n = 7,293 participants (Total N = 14,586). The elimination of differences in baseline characteristics after PSM ensured comparability between the two groups for subsequent analysis.

© 2026 Wu T. et al.
